# Hypercholesterolemia Is an Associated Factor for Risk of Differentiated Thyroid Cancer in Chinese Population

**DOI:** 10.3389/fonc.2020.508126

**Published:** 2021-01-28

**Authors:** Junyu Zhao, Yutian Tian, Jinming Yao, He Gu, Rui Zhang, Huanjun Wang, Lin Liao, Jianjun Dong

**Affiliations:** ^1^Department of Endocrinology and Metabology, The First Affiliated Hospital of Shandong First Medical University & Shandong Provincial Qianfoshan Hospital, Jinan, China; ^2^Department of Endocrinology and Metabology, Shandong Provincial Qianfoshan Hospital, Cheeloo College of Medicine, Shandong University, Jinan, China; ^3^Department of Thyroid & Breast Surgery, The First Affiliated Hospital of Shandong First Medical University & Shandong Provincial Qianfoshan Hospital, Shandong University, Jinan, China; ^4^Department of Thyroid & Breast Surgery, Shandong Provincial Qianfoshan Hospital, Cheeloo College of Medicine, Shandong University, Jinan, China; ^5^Department of Endocrinology and Metabology, Qilu Hospital of Shandong University, Cheeloo College of Medicine, Shandong University, Jinan, China

**Keywords:** differentiated thyroid cancer, thyroid nodule, hypercholesterolemia, total cholesterol, clinical study

## Abstract

**Background:**

Hyperlipidemia has been hypothesized as a risk factor for thyroid cancer. However, the association between hypercholesterolemia and thyroid cancer is unclear, especially in Chinese population without available published data. We conducted this study to investigate the relationship between hypercholesterolemia and differentiated thyroid cancer (DTC) in Chinese population.

**Methods:**

Three thousand seven hundred forty-eight patients were enrolled in the study, including 2,021 DTC patients and 1,727 benign subjects with benign thyroid nodules. Demographic characteristics, medical history, and clinical hematological examination were collected. Stratified analyses of association between hypercholesterolemia and risk of DTC were done. Multivariable logistic regression models were used to estimate the association between hypercholesterolemia and the risk of thyroid nodules being malignant. This study protocol was approved by the ethics committee of Shandong Provincial Qianfoshan Hospital and assigned in ClinicalTrials.gov protocol registration and results system (NCT03006289, https://clinicaltrials.gov/ct2/show/NCT03006289).

**Results:**

The level of serum total cholesterol in patients with DTC is higher than that in benign subjects (*P* < 0.001). After adjusting hypercholesterolemia, age (*P* < 0.001), triglyceride (*P* = 0.003), and thyroid stimulating hormone (TSH) (*P* < 0.001) are found to be confounding factors. The risk of DTC in patients younger than 45 years old is 2.08 times than that of patients older than 45 years old (odds ratio = 0.48, 95% CI (0.38, 0.61), *P* < 0.001). A high TSH level is highly associated with the increased risk of DTC (*P *< 0.001). The multivariable logistic regression analysis revealed that the absence of hypercholesterolemia could reduce the risk of thyroid nodules being malignant (odds ratio = −0.75, 95% CI (−1.39, −0.12), *P* = 0.02). Comparing to the higher level of serum total cholesterol (>5.7 mmol/L), the closer the serum total cholesterol level is to normal (3.17–5.7 mmol/L), the less the risk of thyroid nodules being malignant is, and this difference is statistically significant (odds ratio = −0.67, 95% CI (−1.31, −0.03), *P *= 0.040). However, this difference is not found in the group of patients with lower level of total cholesterol (<3.17 mmol/L, odds ratio = 0.43, 95% CI (−1.22, 2.09), *P *= 0.068), suggesting that hypocholesterolemia is not a protective factor in the risk of thyroid nodules being malignant.

**Conclusions:**

Hypercholesterolemia is an associated factor for risk of DTC in Chinese population.

## Introduction

Thyroid cancer, the most common malignancy in endocrine system, is becoming increasingly prevalent worldwide. In China, the collection of cancer registration data in 2013 showed that the incidence of thyroid cancer in female reached 16.31 per 100,000 and became the fifth most common cancer in female (1). Thyroid cancer is divided into papillary thyroid cancer (PTC), follicular thyroid cancer (FTC), anaplastic thyroid cancer, medullary thyroid cancer, and other rare types. Among them, PTC and FTC are called differentiated thyroid cancer (DTC) and consist of more than 90% of all thyroid cancers (2–4). For better prevention and treatment of DTC, it is particularly necessary to look for the risk factors of the increasing incidence of thyroid cancer.

Nowadays, the known risk factors for thyroid cancer include exposure of radioactive radiation, gender, age, iodine deficiency or excess, and family history of thyroid cancer (5, 6). However, these seem not to be able to fully explain the increased incidence and most of the risk factors, such as gender, age, family history of thyroid cancer, or even genetic mutation are ineluctable. An interesting finding showed that thyroid cancer has been regarded as a high-income lifestyle-associated diseases (1, 7). It was found in the United States that social economic status is strongly associated with thyroid cancer and the richer populations are more likely to develop thyroid cancer (7). Moreover, a survey conducted in China reported that the incidence of thyroid cancer in well-developed cities is four times as risky as undeveloped cities (1). Therefore, more attention has been focused on the preventable and modifiable risk factors, such as factors related to over-nutrition. It has been hypothesized that hyperlipidemia may play a role in the increased incidence of thyroid cancer (8). However, clinical studies on the relationship between hyperlipidemia and risk of thyroid cancer are lacking. Furthermore, which component of lipid plays the key role is still unknown. Therefore, this study aimed to assess the association between hyperlipidemia and DTC in Chinese population, whose diet and lifestyle are different from western population, and explore that which type of hyperlipidemia plays a key role. The influence of other classic risk factors that related to thyroid cancer on the association were also evaluated by stratified analysis.

## Material and Methods

### Study Design and Study Population

In accordance with the STROBE statement, this study with the purpose of estimating the potential relationship between hypercholesterolemia and risk of DTC was performed. Inclusion criteria for this study are as follows: (1) patients who were diagnosed with thyroid nodules by thyroid ultrasonography; (2) patients who underwent a thyroid operation at the Thyroid Surgery Department of the first affiliated hospital of Shandong First Medical University and Shandong Provincial Qianfoshan Hospital (Shandong Provincial Qianfoshan Hospital, Cheeloo College of Medicine, Shandong University) during 1 January 2013 and 30 June 2020; (3) patients who were diagnosed with PTC, FTC, or benign thyroid nodules by a surgical pathology; (4) clinical data can be collected in the electronic medical records system. Participants were excluded based on the following criteria: (1) foreigners except Chinese; (2) a history of thyroid surgery; (3) a history of radioactive iodine therapy; (4) pregnancy or breast-feeding women. Totally, 3,748 patients, diagnosed with thyroid nodules by thyroid ultrasonography and underwent a thyroidectomy, recruited in this study. Of these, 2,021 patients were diagnosed with differentiated thyroid cancer by a pathological examination. The remaining 1,727 patients were diagnosed with benign thyroid nodules. All experiments were performed in accordance with relevant guidelines and protocols. This study protocol was approved by the ethics committee of Shandong Provincial Qianfoshan Hospital and assigned in ClinicalTrials.gov protocol registration and results system (NCT03006289). No sex-based or racial/ethnic-based differences were present. All experiments were performed in accordance with relevant guidelines and protocols.

### Data Collection

Demographic characteristics (sex, age, height, and body weight), history of chronic disease (diabetes, hyperlipidemia, hypertension, hyperthyroidism, hypothyroidism, Hashimoto thyroiditis), long-term medication use (hypoglycemic drugs, hypolipemic agents, hypotensive drugs, antithyroidism drug, thyroxine), and characteristics of each patient’s tumor were collected in the electronic medical records system and recorded using a standardized questionnaire. Clinical hematological examination including fasting plasma glucose (FPG), glycosylated hemoglobin (HbA1c), triglyceride (TG), total cholesterol (TC), high-density lipoprotein-cholesterol (HDL-C), low-density lipoprotein-cholesterol (LDL-C), uric acid (UA), sialic acid, free triiodothyronine (FT3), free thyroxine (FT4), thyroid stimulating hormone (TSH), thyroid peroxidase antibody (TPOAb), thyroglobulin antibody (TgAb), and thyroglobulin (Tg) were also collected in the electronic medical records system. FT3, FT4, TSH, TPOAb, TgAb, and Tg were tested by electrochemical luminescence method. The rest laboratory testing were tested by Roche automatic analyzer. Body mass index (BMI) was determined by dividing weight in kilograms by height meters squared (kg/m^2^) and it was collected to as far back as 2 years prior to the thyroidectomy and as recently as the most recently. All the laboratory testing was tested before the thyroid surgery. Hypercholesterolemia was defined by a fasting serum total cholesterol exceeds the normal limit (total cholesterol >5.7 mmol/L, or a history of hypercholesterolemia).

### Statistical Analysis

Continuous variables with normally distribution were presented as means and standard deviations (SD) and assessed by the Mann-Whitney test. Continuous variables with none normally distribution was presented as median and inter-quartile range and assessed by the Z test. Categorical variables were presented as percentages and frequencies, and the chi-square test was used for categorical variables. The association of hypercholesterolemia and risk of DTC was assessed with the use of multivariable logistic regression models. For further analysis, serum total cholesterol was included as a continuous variable in the model. We also calculated the unadjusted and adjusted odds ratio (OR) with 95% CI. Multivariable logistic regression model for age, sex, systolic blood pressure (SBP), TG, FPG, sialic acid, UA and TSH, TPOAb, TgAb, and BMI were adjusted in different models. We conducted the stratified analyses according to age, sex, UA, plasma sialic acid, TG, FPG, TPOAb, TgAb, TSH, and hypertension [both traditional and new guide (9)]. The statistical analysis was performed by Statistical Package for Social Sciences Version 19.0 software (SPSS Inc., Chicago, IL, USA) and two-side *P* values less than 0.05 indicate statistical significance.

## Results

### Characteristics of the Subjects

There were 2,021 DTC patients and 1,727 subjects diagnosed with benign thyroid nodules enrolled in this study. **Table 1** shows the main characteristics of each group as well as their anthropometric measurements. There were statistically significant differences in age (*P* < 0.001), BMI (*P* = 0.003), SBP (*P* = 0.041), TG (*P* = 0.001), TC (*P* < 0.001), HDL-C (*P* = 0.017), UA (*P* = 0.008), sialic acid (*P* < 0.001), FT3 (*P* = 0.043), Tg (*P* < 0.001), hyperlipidemia history and HT history between the DTC group and benign group. Patients with DTC were younger than the benign subjects (mean age, 47.96 *vs.* 53.93 years, *P* < 0.001). The BMI in DTC group was higher than that in benign group (mean BMI, 25.18 *vs.* 24.63 kg/m^2^), and the difference was statistically significant (*P* = 0.003). Additionally, patients with DTC had a significantly higher level of serum lipid, including TG (*P* = 0.001), TC (*P* < 0.001) and HDL-C (*P* = 0.017), than that in patients with benign thyroid nodules. Twenty-one patients in DTC group and 17 patients in benign group took lipid-lowering therapy.

**Table 1:** Main characteristics of the study population*.

Variable	Patients with DTC (N = 2,021)	Benign subjects (N = 1,727)	*P* value
Age, year	50 (17.0)	49 (16.0)	<0.001
Female sex, N (%)	1,498 (74.1%)	1,300 (75.3%)	0.419
BMI, kg/m^2^	25.00 (4.4)	24.20 (4.4)	0.003
SBP, mmHg	128 (21.0)	130 (19.0)	0.041
DBP, mmHg	77 (15.0)	77 (16.0)	0.096
FPG, mmol/L	5.18 (0.84)	5.21 (0.84)	0.174
HbA1c, %	6.25 (1.25)	6.00 (1.25)	0.271
TG, mmol/L	1.16 (0.84)	1.04 (1.00)	0.001
TC, mmol/L	4.67 (1.34)	4.67 (1.27)	<0.001
HDL-C, mmol/L	1.28 (0.43)	1.34 (0.40)	0.017
LDL-C, mmol/L	2.71 (1.10)	2.69 (0.97)	0.125
UA, umol/L	273 (101.2)	267 (97.15)	0.008
Sialic acid, mg/dl	60.7 (11.10)	61.7 (12.42)	<0.001
FT3, pmol/L	4.92 (0.88)	5.00 (0.87)	0.043
FT4, pmol/L	16.88 (3.47)	16.76 (3.30)	0.780
TSH, uIU/ml	1.97 (1.50)	1.59 (1.45))	0.221
TPOAb, IU/ml	15.02 (16.42)	14.11 (15.46)	0.543
TgAb, IU/ml	19.99 (59.78)	18.03 (24.48)	0.055
Tg, ng/ml	17.11 (33.40)	40.63 (155.28)	<0.001
Diabetes history, N (%)	178 (8.8%)	168 (9.7%)	0.336
Hyperlipidemia history, N (%)	295 (14.6%)	163 (9.4%)	<0.001
Hypertension history, N (%)	759 (37.6%)	601 (34.8%)	0.082
Hyperthyroidism history, N (%)	51 (2.5%)	44 (2.5%)	0.962
Hypothyroidism history, N (%)	19 (0.9%)	25 (1.4%)	0.172
HT history, N (%)	463 (22.9%)	291 (16.9%)	<0.001
Smoking history, N (%)	118 (3.1%)	123 (3.3%)	0.124
Drinking history, N (%)	122 (3.2%)	101 (2.7%)	0.836

*Values are median (interquartile range) or number (%). P values were calculated by comparing characteristics between two groups. BMI, body mass index; SBP, systolic blood pressure; DBP, diastolic blood pressure; FPG, fasting plasma glucose; TG, triglyceride; TC, total cholesterol; HDL-C, high-density lipoprotein-cholesterol; LDL-C, low-density lipoprotein-cholesterol; UA, uric acid; FT3, free triiodothyronine; FT4, free thyroxine; TSH, thyroid stimulating hormone; TPOAb, thyroid peroxidase antibody; TgAb, thyroglobulin antibody; Tg, thyroglobulin; HT, Hashimoto thyroiditis.

### Hypercholesterolemia and Serum Total Cholesterol

**Figure 1:**
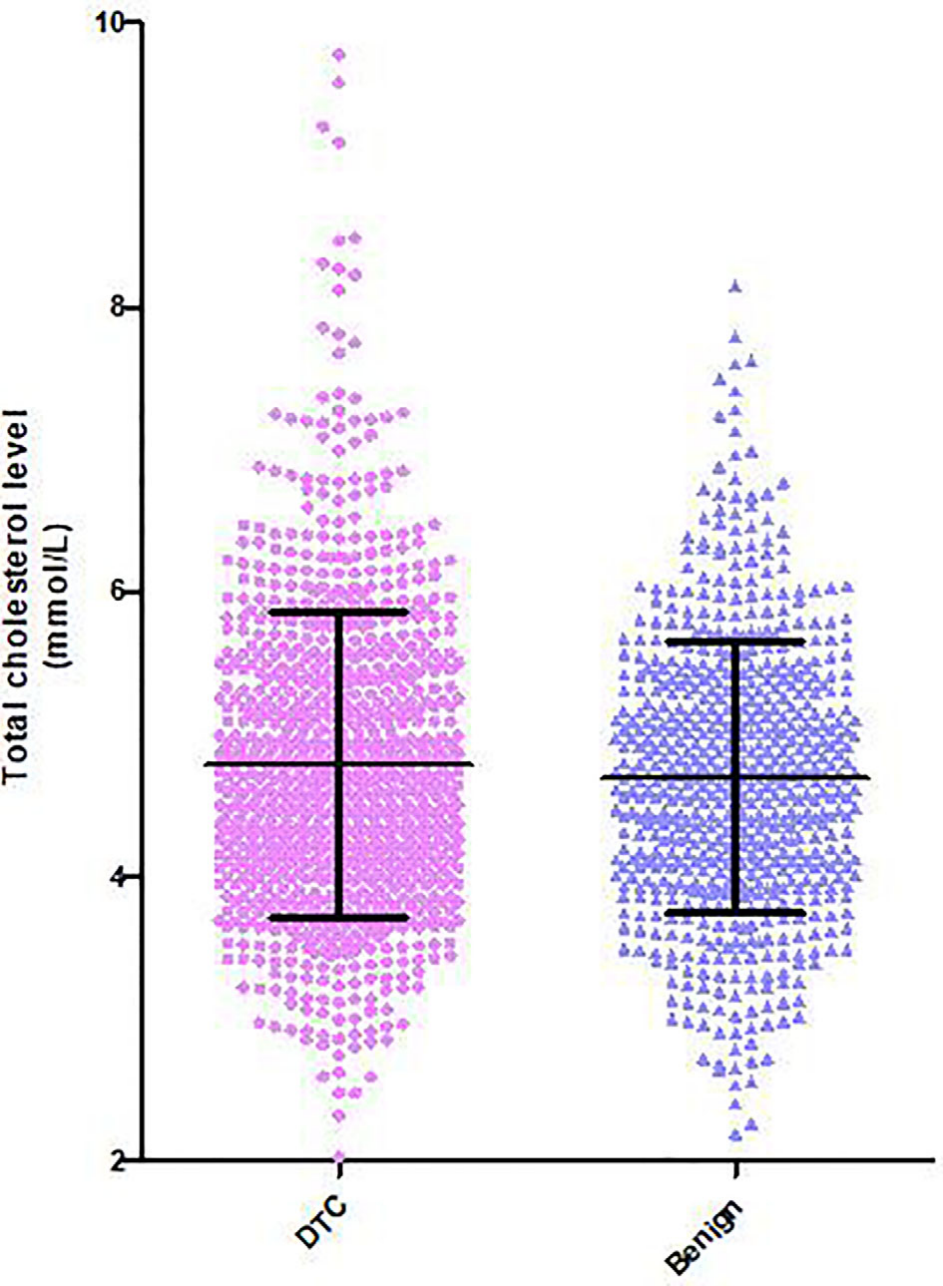
Distribution of serum total cholesterol levels in patients with DTC and benign thyroid nodules. Serum total cholesterol in the DTC group was significantly higher than that in patients without thyroid carcinoma.

Hypercholesterolemia was defined as serum total cholesterol levels higher than 5.7 mmol/L or patients who had been diagnosed as hypercholesterolemia before and were being treated with lipid-lowering drugs. Serum total cholesterol was detected in 253 (12.5%) of the 2,021 DTC patients and in 638 (36.9%) of the 1,727 benign subjects. The mean serum total cholesterol level in the DTC group was significantly higher than that in the benign group (4.79 ± VS. 4.69 mmol/L, *P* < 0.001, **Figure 1**).

### Relationship Between Hypercholesterolemia and Risk of DTC

The stratified analysis of the association between hypercholesterolemia and the risk of DTC were done and showed in **Table 2**. The result of stratified analysis showed that after adjusting hypercholesterolemia, age (*P* < 0.001), TG (*P* = 0.003) and TSH (*P *< 0.001) are found to be confounding factors. The risk of DTC in patients younger than 45 years old is 2.08 times than that of patients older than 45 years old [OR = 0.48, 95% CI (0.38, 0.61), *P* < 0.001]. Furthermore, a statistical association between hypercholesterolemia and risk of DTC was revealed in patients with TG higher than 1.39 mmol/L [OR = 1.52, 95% CI (1.17, 1.97), *P* = 0.002]. A high TSH level is highly associated with the increased risk of DTC both when TSH is grouped by trisected and if TSH is higher than 2 uIU/ml (*P* < 0.001). The risk of DTC was 53% elevated in patients with TSH ≥2.00 uIU/ml than patients with TSH <2.00 uIU/ml [OR = 1.53; 95% CI (1.22, 1.90), *P* < 0.001].

**Table 2:** Association between hypercholesterolemia and risk of DTC according to baseline characteristics.

Subgroup	Number of participants		OR	95% CI		*P* value	*P* *
				Low	High		
Age, year (≥45 years)							<0.001
<45	432		–	–	–	–	
≥45	1,091		0.48	0.38	0.61	<0.001	
Sex							0.425
Female	1,129		–	–	–	–	
Male	394		0.91	0.72	1.15	0.425	
UA, umol/L (Trisection)							0.094
<242	501		–	–	–	–	
≥242, <306	501		1.09	0.85	1.4	0.495	
≥306	508		1.32	1.02	1.69	0.033	
Sialic acid, mg/dl (Trisection)							0.480
<59.5	492		–	–	–	–	
≥59.5, <67.2	495		1.05	0.82	1.36	0.685	
≥67.2	504		0.90	0.70	1.17	0.435	
TG, mmol/L (Trisection)							0.003
<0.9	504		–	–	–	–	
≥0.9, <1.39	510		1.05	0.82	1.34	0.705	
≥1.39	509		1.52	1.17	1.97	0.002	
FPG, mmol/L (Trisection)							0.060
<4.93	492		–	–	–	–	
≥4.93, <5.46	514		0.74	0.57	0.95	0.019	
≥5.46	510		0.82	0.63	1.05	0.119	
TPOAb, IU/ml (Trisection)							0.698
<12.19	449		–	–	–	–	
≥12.19, <20.19	460		1.12	0.86	1.46	0.397	
≥20.19	420		1.06	0.81	1.38	0.692	
TgAb, uIU/ml (Trisection)							0.083
<13.61	312		–	–	–	–	
≥13.61, <29.37	415		0.97	0.72	1.30	0.820	
≥29.37	385		1.31	0.96	1.77	0.086	
TSH, uIU/ml (Trisection)							<0.001
<1.42	446		–	–	–	–	
≥1.42, <2.37	454		1.83	1.40	2.39	<0.001	
≥2.37	452		1.89	1.45	2.47	<0.001	
TSH, uIU/ml (≥2.00 uIU/ml)							<0.001
<2.00	748		–	–	–	–	
≥2.00	604		1.53	1.22	1.90	<0.001	
Hypertension (classic definition: SBP ≥140 mmHg or DBP ≥90 mmHg)							0.208
No	1,094	–		–	–	–	
Yes	407	1.16		0.92	1.47	0.208	
Hypertension (2017 AHA definition: SBP ≥130 mmHg or DBP ≥80 mmHg)							0.212
No	646	–		–	–	–	
Yes	855	0.88		0.71	1.08	0.212	

OR, odds ratio; CI, confidence interval; UA, uric acid; TG, triglyceride; FPG, fasting plasma glucose; TPOAb, thyroid peroxidase antibody; TgAb, thyroglobulin antibody; TSH, thyroid stimulating hormone; SBP, systolic blood pressure; DBP, diastolic blood pressure.

*P value for interaction.

Multivariable logistic regression analysis evaluating the association between serum total cholesterol and risk of DTC was shown in **Table 3**. The multivariate logistic regression analysis showed a significantly association between hypercholesterolemia and risk of DTC in whole participants [OR = −0.36; 95% CI (−0.64, −0.081), *P*=0.012]. After adjusting for a variety of possible confounders (Model I was adjusted for age, sex, SBP, TG, FPG, sialic acid, UA, and TSH. Model II was further adjusted for TPOAb, TgAb, Tg, and BMI), the correlation still remained that the absence of hypercholesterolemia could reduce the risk of thyroid nodules being malignant [Model I: OR = −0.54, 95% CI (−0.87, −0.21), *P* = 0.001; Model II: OR = −0.75, 95% CI (−1.39, −0.12), *P* = 0.02]. Thus, patients with hypercholesterolemia had a 1.33-fold (model II: 1/0.75) higher risk of DTC than those without hypercholesterolemia. Patients who took a lipid-lowering therapy were not enrolled to analysis in the following subgroup analysis. Serum total cholesterol is divided into three groups: low (<3.17 mmol/L), normal (3.17–5.7 mmol/L), and high (>5.7 mmol/L) by the normal reference range. Comparing to the high group (>5.7 mmol/L), the closer the serum total cholesterol level is to normal (3.17–5.7 mmol/L), the less risk of thyroid nodules being malignant, and this difference is statistically significant in model II [OR = −0.67, 95% CI (−1.31, −0.03), *P* = 0.040]. However, this difference is not found in the group of patients with lower level of total cholesterol [<3.17 mmol/L, OR = 0.43, 95% CI (−1.22, 2.09), *P* = 0.068], suggesting that hypocholesterolemia is not a protective factor in the risk of thyroid nodules being malignant.

**Table 3:** Multivariable logistic regression analysis evaluating the association between serum total cholesterol and risk of DTC.

Variable	Model	Number of participants	Classification	OR	(95% CI)	*P* value
Hypercholesterolemia	Crude	1,523		−0.36	−0.64, −0.08	0.012
	Model I	1,301		−0.54	−0.87, −0.21	0.001
	Model II	400		−0.75	−1.39, −0.12	0.02
Total cholesterol	Crude	1,523	Low	−0.54	−1.08, 0.01	0.054
			Normal	−0.35	−0.63, −0.06	0.018
			High	–	–	–
	Model I	1,301	Low	−0.56	−1.23, 0.10	0.097
			Normal	−0.51	−0.84, −0.17	0.003
			High	–	–	–
	Model II	400	Low	0.43	−1.22, 2.09	0.068
			Normal	−0.67	−1.31, −0.03	0.040
			High	–	–	–

Model I was adjusted for age, sex, SBP, TG, FPG, sialic acid, UA, and TSH. Model II was further adjusted for TPOAb, TgAb, Tg, and BMI. SBP, systolic blood pressure; TG, triglyceride; FPG, fasting plasma glucose; UA, uric acid; TSH, thyroid stimulating hormone; TPOAb, thyroid peroxidase antibody; TgAb, thyroglobulin antibody; Tg, thyroglobulin; BMI, body mass index

## Discussion

In this study, we found that serum total cholesterol levels were significantly associated with risk of DTC in Chinese population. Subjects with hypercholesterolemia had a 1.33-fold higher risk of DTC than those without hypercholesterolemia. However, lower level of serum total cholesterol cannot be a protective factor in the risk of thyroid nodules being malignant.

Cholesterol, an essential component of cellular membrane and the precursor of bile acid and steroid hormones, is essential for cell proliferation. Moreover, it has been reported that cholesterol may induce an increase of tumor angiogenesis and proliferation, and a decreased apoptosis of tumor cells (10). Both increased intake and accelerated synthesis of cholesterol were found in the proliferation of malignant tumor cells (11, 12). Increasing studies indicated that high level of cholesterol is associated with various kinds of tumors, such as breast cancer (13, 14), gastric cancer (15), colorectal cancer (16, 17), prostate cancer (18, 19), and renal cancer (20). Cholesterol is the key component of lipid rafts in lipid biomolecules and it can affect the transduction of signal pathway by regulating the structure of lipid rafts. Recently, some studies indicated that lipid raft which was rich in cholesterol took part in the signal pathway of growth factor receptor in tumor cells and abnormal highly cholesterol could break the balance of lipid and protein, thus inducing an abnormal pathological signal and causing the carcinogenesis.

LDL-C and HDL-C are the main forms of cholesterol that exist in the blood and nearly 75% of cholesterol is present in the form of LDL-C. It has been reported that higher levels of LDL-C and TG were considered as the risk factor of cancer (21). A higher level of LDL-C might be associated with hematological malignant tumor (22) and a lower level of HDL-C (≤20 mg/dl) could lead to a 6.5-fold increased risk of cancer (23). Notably, LDL-C has been confirmed to be able to induce a proliferation and metastasis of breast cancer by activating ErbB2 pathway and reducing the expression of adhesion molecule (14). In addition, another potential mechanism was that increased level of LDL-C and LDL-C/HDL-C contributed to the metastasis of lymph node. HDL-C has an anti-tumor efficiency by inhibiting O_2_, H_2_O_2_, and other hydroxyl radicals (24). Moreover, HDL-C was supposed to inhibit the proliferation and growth of tumor by inhibiting the expression of tumor necrosis factor-α and interleukin-6, whereas it induced the apoptosis of tumor cells by promoting the expression of interleukin-10 (25). Interestingly, the expression of LDL-C receptor in tissues of breast cancer was higher than that in normal tissue, which might indicate that LDL-C was active in breast cancer cells, which might be one of the mechanisms (26).

As the incidence of thyroid cancer has increased dramatically worldwide, a large number of clinical studies and mechanistic experiments are devoted to find the potential risk factors that caused such change (27–32). However, if there is any association between hyperlipidemia and thyroid cancer remains a question. Our research focused on a large sample size of population with thyroid nodule(s) and each patient has undergone thyroid nodule surgery with the pathological confirmation. Secondly, a comprehensive clinical data is collected in the current research, including thyroid function, thyroid related antibodies, and even detailed disease history. Thirdly, not only both male and female data but also a wide range of different age groups from 12 to 86 years old, were included. Thus the founding of our study can be extended throughout both sex and to a relatively wider population. Unfortunately, there are some limitations in our study: (1) Not all of the 3,748 patients have the results of lipid measurements or other parameters that shorten the sample size, which might influence the power of estimation. (2) Body weight was collected as far back as 2 years prior to the thyroid surgery and as recently as the most recent that might cause the recall bias which could not be avoidable. These limitations should be considered in the further studies.

Cholesterol metabolism plays an important role in the occurrence of tumor development, invasion, and metastasis. Nowadays, there were many studies regarding the relationship between cholesterol metabolism and tumors, but limited studies paid attention to the thyroid cancer. Notably, this is the first clinical study demonstrating that hypercholesterolemia is an associated factor for risk of DTC in Chinese population. Furthermore, the mechanism of cholesterol and its components on the complex pathophysiological processes of these malignant tumors still unknown, and that call for further investigations. Based on the previous research and current study, we speculate that cholesterol may play a role in the malignancy risk of thyroid nodule.

## Conclusions

In conclusion, hypercholesterolemia is an associated factor for risk of DTC in Chinese population. A prospective and randomized controlled trial of cholesterol lowering therapy and further basic researches on the mechanism of hypercholesterolemia and risk of DTC are needed to confirm the results.

## Data Availability Statement

All datasets generated for this study are included in the article/supplementary materials.

## Ethics Statement

The studies involving human participants were reviewed and approved by Shandong Provincial Qianfoshan Hospital. Written informed consent to participate in this study was provided by the participants’ legal guardian/next of kin, or by the participants themselves if they were of legal consenting age.

## Author Contributions

JZ, LL, and JD conceived of and designed the study. JZ, YT, JY, HG, RZ, and HW collected and analyzed the data. JZ wrote the paper. LL and JD supervised the whole study and revised the manuscript. All authors contributed to the article and approved the submitted version.

## Funding

This work was supported by the Projects of Medicine and Health Science Technology Development Program in Shandong Province (grant number 2016WS0499, 2014WS0103), National Natural Science Foundation of China Grants (grant number 81570742, 81670757, 81770822) Shandong Provincial Natural Science Foundation of China Grants (grant number ZR2016HQ26, ZR2019PH025), and Grant for the development of science and technology of Jinan City (grant number 201602172). They support the study design; the collection, analysis, and interpretation of data; the writing of the report; and the decision to submit the article for publication.

## Conflict of Interest

The authors declare that the research was conducted in the absence of any commercial or financial relationships that could be construed as a potential conflict of interest.
